# Ixazomib-Lenalidomide-Dexamethasone for the Treatment of Relapsed/Refractory Multiple Myeloma: A Hungarian Real-World Analysis

**DOI:** 10.3390/jcm15010286

**Published:** 2025-12-30

**Authors:** Hermina Sánta, Laura Regáli, László Váróczy, Virág Szita, Ádám Wiedemann, Lóránt Varju, László Rejtő, Norbert Sándor Bartha, Dorottya Máté, András Masszi, Márk Plander, Szabolcs Kosztolányi, Alizadeh Hussain, Piroska Pettendi, Ildikó Istenes, Árpád Szomor, Péter Reményi, Tamás Masszi, Gergely Varga, Gábor Mikala

**Affiliations:** 1 3rd Department of Internal Medicine, Hematology Fejér County University Hospital, 8000 Székesfehérvár, Hungary; dr.santa.hermina@gmail.com (H.S.); aszomor2@mail.fmkorhaz.hu (Á.S.); 2 National Institute for Haematology and Infectious Diseases, South-Pest Central Hospital, 1097 Budapest, Hungary; laura.julia.regali@gmail.com (L.R.); premenyi@dpckorhaz.hu (P.R.); 3 Department of Hematology, Institute for Medicine, Faculty of Medicine, University of Debrecen, 4032 Debrecen, Hungary; laszlo.varoczy@gmail.com; 4 Department of Internal Medicine and Hematology, Semmelweis University, 1088 Budapest, Hungary; virag.szita@gmail.com (V.S.); wiedemannadam@gmail.com (Á.W.); masszi.tamas@semmelweis.hu (T.M.); vargager@gmail.com (G.V.); 5 Department of Hematology, Szabolcs-Szatmár-Bereg County Hospital, 4400 Nyíregyháza, Hungary; varjulorant@gmail.com (L.V.); lrejto@med.unideb.hu (L.R.); drbnorbs@gmail.com (N.S.B.); 6 Department of Hematology, National Institute of Oncology, 1122 Budapest, Hungary; mate.dorottya@oncol.hu (D.M.); amasszi@gmail.com (A.M.); 7 Department of Hematology and Hemostaseology, Vas County Markusovszky University Hospital, 9700 Szombathely, Hungary; planderm@yahoo.com; 8 First Department of Internal Medicine, University of Pécs-Clinical Centre, 7624 Pécs, Hungary; kosztolanyi.szabolcs@pte.hu (S.K.); alizadeh.hussain@gmail.com (A.H.); 9 Department of Hematology, Jász-Nagykun-Szolnok County Géza Hetényi Hospital, 5000 Szolnok, Hungary; piri.pet@gmail.com; 10 Department of Internal Medicine and Oncology, Semmelweis University, 1083 Budapest, Hungary; istildi78@gmail.com

**Keywords:** multiple myeloma, relapsed, ixazomib, biochemical progression

## Abstract

**Background/Objectives**: Despite therapeutic advances, managing relapsed/refractory multiple myeloma (RRMM) remains challenging. For patients with frailty, comorbidities, mobility limitations, or when treatment preference and drug accessibility are key considerations, the all-oral ixazomib–lenalidomide–dexamethasone (IRd) regimen offers a practical alternative. **Methods**: We performed a multicenter retrospective study of RRMM patients treated with IRd in Hungary between 1 January 2020 and 30 June 2025. **Results**: The median age at treatment initiation was 73.7 years. Treatment was initiated for clinical progression in 38.2%, biochemical progression in 53.3%, and for intolerance or toxicity of prior therapy in 8.6%. Median progression-free survival (PFS) was 18.7 months, and median overall survival (OS) was 34.7 months. Patients treated at biochemical progression had significantly longer PFS than those treated at clinical progression (24.3 vs. 15.6 months; *p* = 0.004), with additional benefit when IRd was initiated owing to intolerance or toxicity of previous therapy (*p* = 0.04). In the second-line setting, median PFS was 24.5 months, and median OS was not reached. Adverse events occurred in 68.3% of patients; dose reductions were required in 18.4%, and 21.6% discontinued treatment because of intolerance or toxicity. Most common toxicities were neutropenia (32.9%), thrombocytopenia (27.6%), diarrhoea (25%), peripheral neuropathy (25.3%), and infections (22.4%). **Conclusions**: IRd initiation at biochemical progression was associated with superior PFS compared with treatment at clinical progression. When compared with a recent Hungarian multicenter cohort treated with second-line daratumumab, lenalidomide, and dexamethasone, outcomes with IRd are not significantly inferior (36-month OS calculated from 2nd line treatment initiation: 65.5% for DRd vs. 60% in our cohort; *p* = 0.56). These real-world data support IRd as an effective, convenient, all-oral option for appropriately selected RRMM patients.

## 1. Introduction

Multiple myeloma is a neoplastic disorder of plasma cells and ranks as the second most common hematologic malignancy [1]. The management of relapsed and/or refractory multiple myeloma (RRMM) remains challenging despite the expanding therapeutic landscape. A key unresolved question is the optimal timing of treatment initiation in patients with biochemical progression, as several studies have reported inferior outcomes when therapy is deferred until clinical relapse [2,3]. Early intervention at biochemical progression may allow better tailoring of treatment and prevention of irreversible end-organ damage [1]. In contrast, in slowly progressive disease, intervention at an asymptomatic stage may lead to overtreatment, heightened toxicity, and cost [4]. Although multiple innovative treatment modalities—including next-generation immunomodulatory drugs, proteasome inhibitors, monoclonal antibodies, antibody–drug conjugates, bispecific antibodies, and CAR T-cell therapies—have broadened the therapeutic armamentarium for RRMM [5], real-world practice patterns exhibit substantial variability across regions and centers [6]. There is no consensus on which treatment option should be used in a specific patient group beyond consideration of prior therapy sensitivity. In routine clinical practice, treatment choice is often guided by anticipated efficacy, tolerability, comorbidities, patient preference, logistical concerns, and local drug availability [7]. Consequently, clinical scenarios arise in which novel agents with the highest efficacy are impractical or unsuitable. For frail patients, those with significant comorbidities, mobility limitations, or where treatment preference and accessibility are key considerations, an all-oral regimen may represent a particularly attractive option. In the pivotal TOURMALINE-MM1 trial, IRd significantly prolonged progression-free survival compared with placebo–Rd (20.6 vs. 14.7 months) [8]. Although in routine clinical practice the results of clinical trials are often not reproducible, with IRD, comparable outcomes were observed in several real-world prospective and retrospective studies [8,9,10,11,12,13]. Most prevalent toxicities of ixazomib include infections, thrombocytopenia, neutropenia, peripheral neuropathies, gastrointestinal symptoms, and rash [8]. However, the majority of patients tolerate the IRd protocol well, making it a reliable choice for frail patients. Building on a prior Hungarian real-world analysis conducted between 2015 and 2017 [13], the present study aims to delineate which patient groups derive the most significant benefit from IRd in the contemporary therapeutic era, particularly in the context of widely available daratumumab-based regimens. With data available from a recent real-world Hungarian study on daratumumab, lenalidomide, and dexamethasone (DRd) in the second-line setting [14], we aimed to provide data to support the decision between these two protocols, often used in the second-line treatment.

## 2. Materials and Methods

We conducted a multicenter retrospective cohort study of RRMM patients treated with an ixazomib-based regimen across Hungarian centers from 1 January 2020 to 30 June 2025. The patient cohort was based on hospital pharmacy records of ixazomib prescriptions; all consecutively treated patients were included in the study, and data were collected uniformly across all centres. Regimens combining ixazomib with daratumumab, were excluded from this study (Supplementary Figure S1). Clinical data were retrospectively extracted from electronic medical records, and all patients provided consent for data collection. The study was approved by the Central Ethics Committee of Hungary and conducted in accordance with the Declaration of Helsinki. Eligible patients had received at least one prior line of therapy before starting an ixazomib-containing regimen. The primary endpoint of our analysis was progression-free survival (PFS), defined as the interval from treatment initiation to disease progression or death. The secondary endpoint was overall survival (OS), defined as the time from ixazomib treatment initiation to death from any cause. Response and survival outcomes were assessed according to International Myeloma Working Group (IMWG) criteria [15]. High-risk cytogenetics included t(4;14), t(14;16), 1q gain/amplification, and del(17p); patients harboring ≥2 of these abnormalities were classified as ultra–high risk. Fisher’s exact test was used for comparisons of categorical variables. Continuous variables were analyzed using the Mann–Whitney U test and Spearman correlation. PFS and OS were estimated using the Kaplan–Meier method and covariates were compared using Cox regression to estimate hazard ratios (HRs) and 95% confidence intervals (CIs). Statistical analyses were performed using IBM SPSS Statistics version 30.0.0.0.

## 3. Results

### 3.1. Patients

A total of 152 patients from nine Hungarian centers were included in the analysis. Baseline characteristics are summarized in Table 1. The most common M-protein type was IgG κ or λ (42.1% and 20.4% respectively), followed by κ or λ free light chain disease (7.9% and 3.3% respectively); the remaining 26.3% had other isotypes. The median follow-up from diagnosis was 66.3 months (range, 2.3–271.1), and the median age at ixazomib initiation was 73.7 years. Cytogenetic information was available for 114 patients. High-risk abnormalities—defined as t(4;14), t(14;16), 1q gain/amplification, or del(17p)—were present in 34.2% at any time from diagnosis to the start of ixazomib therapy. Seventeen patients (11.2%) met criteria for ultra–high-risk disease.

Prior exposure to lenalidomide, daratumumab, and pomalidomide was observed in 59.2% (*n* = 90), 8.6% (*n* = 13), and 5.3% (*n* = 8), respectively, with refractoriness to lenalidomide in 13.2% (*n* = 20), to daratumumab in 5.3% (*n* = 8), and pomalidomide in 2.6% (*n* = 4) (Table 1). Among daratumumab-exposed patients, IRd was administered after 1, 2, 3, or ≥4 prior lines of therapy in 38%, 23.8%, 23.8%, and 14.4% of cases, respectively. Of those refractory to daratumumab, 75% were triple-refractory (to a proteasome inhibitor, lenalidomide, and daratumumab), while the remaining 25% were additionally refractory to pomalidomide. Prior to autologous stem cell transplantation (ASCT) was performed in 34.9% (*n* = 53) of patients, of whom 29 received subsequent maintenance therapy. There was no statistically significant difference in PFS between patients who underwent ASCT and those who did not (*p* = 0.5). However, the interval from diagnosis to ixazomib initiation was approximately twice as long in autotransplanted patients (median 4.8 vs. 2.7 years). Second-line patients had a median age comparable to the overall cohort and included a higher proportion of lenalidomide-naïve individuals. Only one second-line patient was refractory to daratumumab (Table 1).

### 3.2. Treatment Characteristics

The median interval from diagnosis to initiation of ixazomib therapy was 45.6 months, and the median treatment duration was 8 months. Ixazomib-based therapy was initiated after 1, 2, 3, or ≥4 prior lines in 43.4%, 32.9%, 16.4%, and 7.2% of patients, respectively. Most received IRd (89.5%), while 10.5% were treated with off-label individual combinations, including ixazomib–dexamethasone or ixazomib–lenalidomide doublets, or triplets incorporating cyclophosphamide or melphalan instead of lenalidomide. Ixazomib was administered at 4 mg QD in 88.2% of cases and at 3 mg in 11.8%. Lenalidomide dosing was 25 mg QD, 15 mg QD, 10 mg, or <10 mg daily (21/28 days) in 34.2%, 10.5%, 39.5%, and 10.8% of patients, respectively. Dexamethasone (or an equivalent methylprednisolone dose) was given weekly at 40 mg, 20 mg, 12 mg, or 8 mg in 24.3%, 47.3%, 12.2%, and 16.2% of patients, respectively. There was no significant association between PFS and ixazomib daily dose (4 mg vs. 3 mg) (*p* = 0.554), lenalidomide daily dose (standard dosing of 25 mg daily vs. reduced doses of 15 mg, 10 mg, or less) (*p* = 0.119), or dexamethasone weekly dosing of 20 or 40 mg vs. reduced doses (*p* = 0.43)

Treatment initiation patterns varied: therapy began for clinical relapse in 38.2%, biochemical progression in 53.3%, and intolerance or toxicity of prior treatment in 8.6%. The latter group primarily consisted of patients experiencing toxicities—most commonly neuropathy—during bortezomib-based therapy, for whom an in-line switch- therapy change in the same drug class- from bortezomib to ixazomib was favored over treatment discontinuation.

### 3.3. Treatment Efficacy

**Figure 1:**
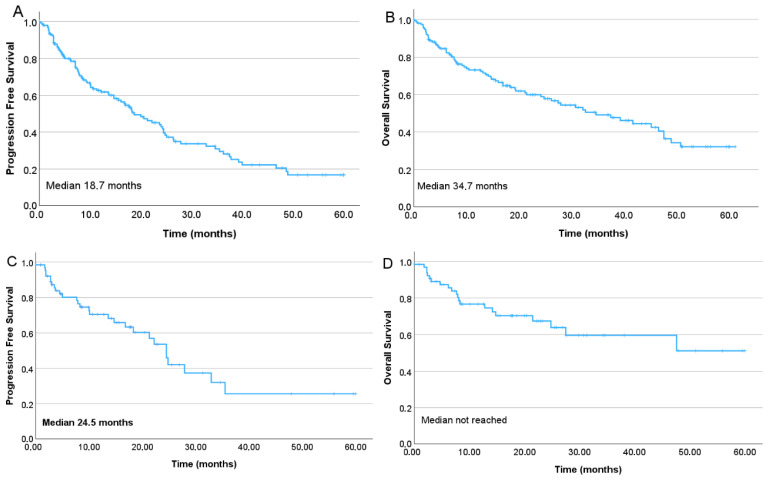
(**A**) Progression-free survival of the entire cohort of IRd-treated myeloma patients; (**B**) Overall survival of the whole cohort; (**C**) Progression-free survival in the second line; (**D**) Overall survival in the second line. Overall survival was calculated from ixazomib treatment initiation.

**Figure 2:**
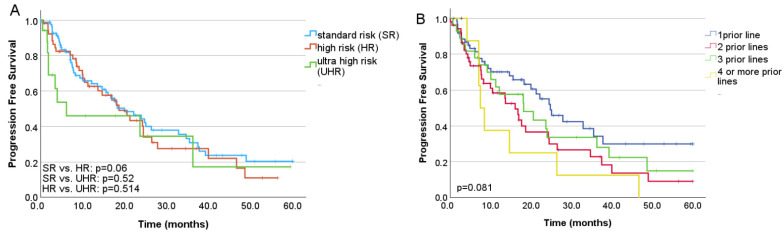
Progression free survival based on (**A**) cytogenetic risk (**B**) number of prior therapy lines.

**Figure 3:**
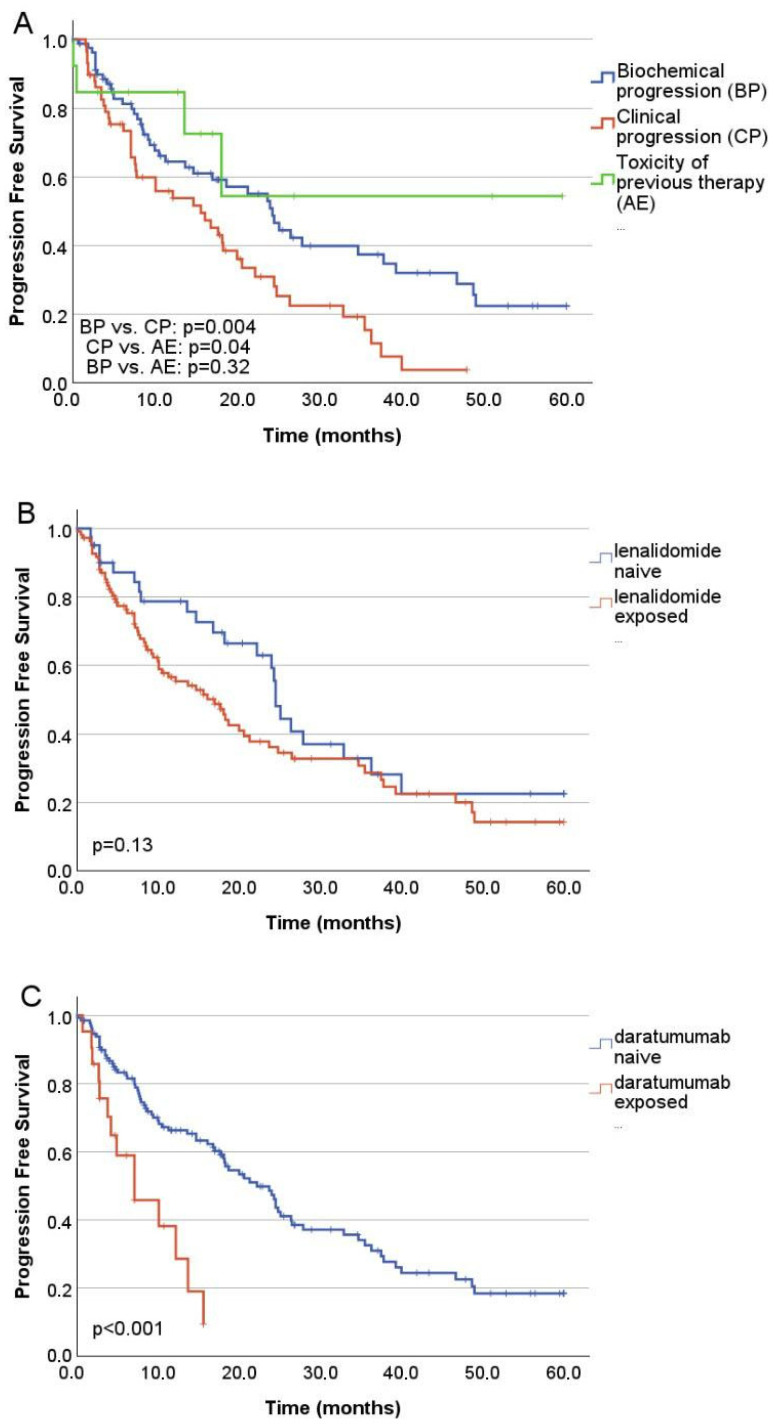
Progression free survival based on (**A**) the reason for treatment initiation; SR—standard risk, HR—high risk, UHR—ultra-high risk, BP—biochemical progression, CP—clinical progression, AE—toxicity of previous therapy. (**B**) prior lenalidomide exposure; (**C**) prior daratumumab exposure.

Overall response rate (ORR) was 67.8%. Median PFS was 18.7 months, and the median overall survival (OS) was 34.7 months (Figure 1A,B). In the second-line subgroup, the estimated median PFS was 24.5 months, while median OS had not yet been reached (Figure 1C,D). The presence of high-risk cytogenetics did not increase the risk of progression, (*p* = 0.073, HR: 1.46, 95% CI: 0.966, 2.208) (Figure 2A), however, cytogenetic data was missing in 25% (*n* = 38) of the patients, and high-risk and ultra-high risk cohorts were rather small. In a multivariate analysis no statistically significant impact on the PFS was found for the ISS stage at diagnosis (*p* = 0.076, HR 1.39, CI 95% 0.95–2.00). Higher number of prior therapy lines was associated with higher risk of progression (*p* = 0.011, HR: 1.43, CI 95%: 1.088, 1.893), lower overall response rates and numerically shorter PFS (One previous line: ORR: 65.1%, PFS: 24.8 months; two previous lines: ORR 67.3%, PFS: 16.1 months; three previous lines: ORR 57.1%, PFS: 18.2 months; four or more previous lines: ORR 33.3%, PFS: 7.5 months) (Figure 2B). Patients treated at biochemical progression achieved significantly longer PFS than those treated at clinical relapse (24.3 vs. 15.6 months; *p* = 0.004). Even more favorable outcomes were observed when ixazomib was initiated due to intolerance or toxicity compared with clinical progression (*p* = 0.04). There was no significant difference in the PFS after switching due to toxicity compared with that due to biochemical progression (*p* = 0.32) (Figure 3A). Of all toxicity-driven in-line switches to ixazomib, 60% occurred in the second line. These patients also initiated ixazomib considerably earlier after diagnosis (median 7.2 months) than those treated at biochemical progression (44.4 months) or clinical progression (52.8 months), while age at ixazomib initiation remained similar across groups (73.9, 73.9, and 75 years, respectively). Notably, the advantage observed in PFS among patients treated at biochemical progression translated into a significant overall survival benefit (45.2 vs. 21.2 months; *p* = 0.018).

Prior lenalidomide exposure did not adversely influence PFS (*p* = 0.13) and risk of progression (*p* = 0.359, HR: 0.81, CI 95%: 0.516, 1.27). However, lenalidomide refractoriness was associated with a trend toward shorter PFS as compared to lenalidomide-naïve patients (*p* = 0.09). (Lenalidomide naïve: ORR: 73.8%, PFS: 24.5 months; lenalidomide exposed: ORR: 62.2% PFS: 16.1 months; lenalidomide refractory: ORR: 50% PFS: 18.7 months) (Figure 3B). Only a small percent of patients were previously exposed to pomalidomide, their risk of progression was not statistically significantly different (*p* = 0.984, HR: 1.00, 95% CI 0.46, 2.21), but their PFS was shorter (*p* = 0.02). Patients previously exposed to daratumumab experienced significantly shorter PFS (*p* < 0.001; respectively), and lower overall response rates (ORR) (daratumumab naïve: ORR 67.2%, PFS: 22.2 months; daratumumab exposed: ORR: 46.2%, PFS: 7.1 months; daratumumab refractory: ORR 12.5%, PFS 7.1 months), their risk of progression increased significantly (*p* = 0.01, HR: 2.135, CI 95%, 1.16, 3.92) (Figure 3C). The best response to ixazomib-based therapy was complete response in 9.9%, very good partial response in 15.1%, partial response in 38.2%, and stable disease/no response in 30.3%; progressive disease occurred in 6.6%. Deeper responses were associated with significantly prolonged PFS (*p* < 0.001). No patient in the daratumumab refractory group reached a better response than PR.

### 3.4. Safety

Dose reductions were required in 18.4% of patients. Adverse events (AEs) were reported in 68.3% of the cohort. The most frequent hematologic toxicities were neutropenia (32.9%, including 9.2% grade 3–4) and thrombocytopenia (27.6%; grade 3–4: 7.3%). Non-hematologic AEs included diarrhoea (25%; grade 3: 6.6%), infections (22.4%; grade 3–4: 11.9% and grade 5: 3.9%), and peripheral neuropathy (25.3%; grade 3: 2.7%). Other AEs occurred in 17.1% of patients. Treatment discontinuation due to intolerance or toxicity was necessary in 21,6% (*n* = 33) of cases, of which 51.5% occurred within the first 60 days of therapy.

## 4. Discussion

While clinical trials tend to focus on the efficacy and safety of a given therapy, in real-world settings, the optimal treatment for individual patients is influenced by several other variables. Geographical, socio-economic, and patient-related factors may limit prolonged parenteral anti-myeloma therapy. All oral therapy, self-administered by the patient, may offer a solution for some of these issues, leading to prolonged treatment and better outcomes [16]. Strict eligibility criteria, which are not met by up to 72% of RRMM patients, lead to substantial differences between patient populations in randomized controlled trials and the real world. Apart from patient-related factors, disease- and treatment-related factors may also influence results in these settings. Thus, comparing clinical trial data with real-world evidence should be undertaken with caution [17]. The TOURMALINE-MM1 trial compared to our cohort evaluated IRd versus placebo-RD in a younger and less advanced population (median age 66 vs. 73 years; ISS stage I: 62.8% vs. 25.7% in the TOURMALINE study and our results, respectively), yet reported a similar outcomes (PFS 20.6 vs. 18.7 months) [8]. Despite differences in patient populations, several observational studies have also demonstrated PFS results broadly consistent with those of TOURMALINE-MM1 [8,9,10,11,12,13]. Compared with our earlier Hungarian real-world analysis of IRd, this cohort achieved a longer PFS, likely reflecting earlier administration of ixazomib (second-line use in 43.4% vs. 27% in the earlier dataset) [12], a shift largely driven by changes in national reimbursement policies over recent years. The INSURE study—an integrated analysis of three global observational datasets (INSIGHT MM, UVEA-IXA, and REMIX)—included 564 IRd-treated patients [18]. Although the median age of the INSURE cohort was markedly lower than in our study (64 vs. 73.7 years), the frail subgroup was similar in age to our population (76 vs. 73.7 years), suggesting that in Hungary, IRD is preferentially selected in an older, more vulnerable population. On the contrary, the INSURE group was actually almost 10 years younger at relapse than the typical myeloma patient is at diagnosis, indicating that here primarily lifestyle considerations led to the choice of ixazomib treatment Our patients also had a longer interval from diagnosis to IRd initiation (45.6 vs. 39.3 months) and a higher incidence of high-risk cytogenetic abnormalities (36.9% vs. 15.3%). Despite these differences, ORR and PFS were comparable (67.8% vs. 64.6% and 18.7 vs. 19.9 months, respectively). Consistent with our observations, in the INSURE pooled analysis, prior proteasome inhibitor or lenalidomide exposure did not negatively impact the effectiveness of IRd; however, outcomes were more favorable in patients who were not refractory to these agents [19]. In a retrospective analysis by Fric et al. of patients treated with IRd, there was no correlation between daratumumab refractoriness alone and survival; however, triple refractory status did significantly worsen outcomes [20]. In our cohort, only a small number of patients were exposed to daratumumab before starting ixazomib. In these patients, daratumumab exposure was associated with significantly reduced PFS. Real-world comparisons of lenalidomide-based regimens have shown that daratumumab achieves the highest ORR and the longest PFS (23.6 vs. 11.6–19.9 months) among all combinations. However, IRd recipients in those analyses were older, less fit, had more advanced disease, and were more heavily pretreated, which may have contributed to the observed advantage [21,22]. Furthermore, outcomes with IRd appear to vary by treatment era. Before EU reimbursement in 2019, ixazomib was mainly accessible through named-patient programs in Hungary [12]. In contrast, after reimbursement, it has been used more often in older, frail, or otherwise DRd- or KRd-ineligible patients, which may partly explain the shifts in real-world outcomes [21]. A recent Hungarian multicenter analysis reported outcomes for second-line DRd across seven institutions [14]. The median PFS from DRd initiation was 22 months, with a 3-year PFS of 45.3%. Compared with our second-line IRd cohort, PFS did not differ significantly (22 vs. 24 months; *p* = 0.531; Supplementary Figure S2A). Median OS from second line was not reached in either group; 3-year OS was 65.5% with DRd and 60% with IRd (*p* = 0.562, Supplementary Figure S2B). Notably, DRd-treated patients were substantially younger (median 65 vs. 73 years), suggesting that clinicians may favor an all-oral regimen for older individuals and reserve parenteral therapy for younger or fitter patients. Apart from the age difference, there are other potential confounders, such as patient characteristics, disease biology, duration of follow-up, and treatment, which may impact outcomes. Those were not accounted for in this cross-trial comparison; thus, it must be interpreted with caution. Randomized-controlled trials are needed for head-to-head evaluation of these regimens, especially in the older, more frail population, for whom a convenient regimen is of importance to remain on the therapy. In our analysis, we did not detect significant differences in PFS among standard-risk, high-risk, and ultra–high-risk cytogenetic subgroups (Figure 2A), consistent with prior evidence that proteasome inhibitor–based regimens can mitigate the adverse impact of high-risk cytogenetics [8,21,22].

A subset of patients underwent an in-class switch from bortezomib to ixazomib due to toxicity, without evidence of progression; this group had markedly superior survival outcomes compared with the overall cohort. A study by Rifkin et al. examined in-class switching of proteasome inhibitors in newly diagnosed multiple myeloma patients and found that transitioning from bortezomib-based therapy to IRd was associated with higher overall response rates, longer treatment duration, and fewer discontinuations. Although our cohort comprised mostly patients with relapsed or refractory disease, the results similarly support the potential benefits of an in-class switch strategy in the absence of progression [16]. Abe et al. evaluated the efficacy and safety of transitioning to IRd following three cycles of parenteral proteasome inhibitor–based therapy in patients who had achieved at least a minor response. Notably, the proportion of patients attaining a complete response or very good partial response increased from approximately 7.5% to 42% following the switch to IRd. These findings suggest that sequential therapy with IRd is a feasible strategy that may prolong proteasome inhibitor–based treatment and thereby improve clinical outcomes [23]. The Myeloma XIV FiTNEss trial, conducted by the United Kingdom Myeloma Research Alliance, randomized newly diagnosed multiple myeloma patients to receive either standard or IMWG frailty score–adjusted IRd dosing. The key distinction between the two arms was the reduced daily lenalidomide dose and the weekly dexamethasone dose for patients classified as unfit or frail based on the IMWG Frailty score, while the ixazomib dose remained. Response rate and MRD negativity at 6 months were similar, and 1 year OS was longer in the frailty-adjusted arm [24]. Although our cohort consisted of RRMM patients who were not exclusively elderly or frail, we also observed no significant differences in outcomes between the various dosing regimens of these agents. Collectively, these findings suggest that frail patients benefit from a more conservative treatment strategy using adjusted IRd dosing. A study by Bao et al. compared induction with IRd vs. ixazomib, pegylated liposomal doxorubicin, and dexamethasone in newly diagnosed elderly and frail patients, and concluded that IRd was associated with a higher response rate and improved health-related quality of life, with relatively low toxicity [25]. The combination of ixazomib, daratumumab, and dexamethasone in frail patients in the Hovon-143 study yielded heterogeneous results, with early treatment discontinuation mainly due to disease progression, toxicity, or early death influencing results negatively [26]. Early treatment cessation was observed at similar rates in other studies, including frail patients [24]. Similarly, in our results, half of all treatment discontinuations occurred in the first 60 days of therapy.

Beyond efficacy, safety, and fitness, patient preference is an essential component of treatment selection, which may influence treatment duration and thereby outcomes. The EASMENT study, which assessed 399 patients (including 206 with relapsed/refractory disease), demonstrated a strong preference for oral therapy among RRMM patients and reported higher hospitalization rates among those receiving parenteral treatments [27]. Whether treatment is initiated at the stage of biochemical progression or deferred therapy until clinical progression may also be influenced by individual patient preferences and values. Some patients prioritize preventing end-organ damage and therefore favor earlier intervention, whereas others may prefer to prolong treatment-free intervals and accept closer monitoring. Our findings underscore a clear survival advantage to initiating therapy at the biochemical rather than the clinical stage. Given high efficacy, greater ease of administration, and the majority of patients’ strong preference for oral regimens, IRd can be beneficial for treating biochemical progression after bortezomib-based regimens, if clinically indicated.

The safety profile in our cohort was consistent with other real-world series and the original clinical trial [8,9,10,11,12,13]. Neutropenia was the most common adverse event in our study, whereas other analyses have reported infections [12] or diarrhea [28] as the predominant toxicity.

Carfilzomib is widely regarded as a more potent proteasome inhibitor; however, a real-world comparison of IRd vs. KRd found no significant difference in ORR or PFS, although KRd yielded deeper responses, IRd was associated with more durable remissions [29]. Another extensive retrospective analysis (*n* = 956; 1:1 IRd vs. KRd) demonstrated substantially higher rates of new-onset arrhythmia and heart failure with carfilzomib [30]. Therefore, in a setting of cardiac comorbidities—a common finding in elderly patients—IRd may be a valuable alternative.

In summary, in a population broadly comparable to those of other real-world cohorts, our findings support the efficacy and tolerability of ixazomib-based regimens. Patients treated at biochemical progression and those switched in-line due to intolerance experienced the most favorable outcomes. While daratumumab-based regimens may confer slightly longer OS in cross-study comparisons, oral IRd remains an attractive option, particularly for older, frail patients or those who prioritize treatment convenience. As anti-CD38-based triplets and quadruplets continue to move into the frontline setting, many patients worldwide will still require well-tolerated second-line options, and our data provide helpful guidance for such decision-making.

## Figures and Tables

**Table 1:** Patient characteristics. Abbreviations: ISS—international staging system; NA—not available; FISH—fluorescent in situ hybridization; FLC—free light chain; ASCT—autologous stem cell transplantation.

	Whole Cohort	Second Line
*n*	152	65
Male/female (%)	66/86 (43.4/56.5)	27/38 (41.5/58.5)
Median age at the start of ixazomib (years) (min.–max.)	73.7 (49.3–90.8)	73.8 (50.8–90.8)
ISS I (%)	39 (25.7)	16 (24.6)
ISS II (%)	51 (33.6)	21 (32.3)
ISS III (%)	47 (30.9)	21 (32.3)
ISS NA (%)	15 (9.9)	7 (10.8)
FISH		
Standard risk	45 (29.6)	20 (30.8)
High risk	52 (34.2)	24 (36.9)
Ultra-high risk	17 (11.2)	4 (6.2)
NA	38 (25)	17 (26.2)
M-protein		
IgG kappa/lambda (%)	64/31 (42.1/20.4)	28/11 (43.1/16.9)
FLC kappa/lambda	12/5 (7.9/3.3)	8/2 (12.3/3.1)
other (%)	40 (26.3)	19 (29.2)
Previous lines		
1	66 (43.4)	
2	50 (32.9)	
3	25 (16.4)	
4 or more	11 (7.2)	
Lenalidomide		
naïve	42 (27.6)	27 (41.5)
exposed	90 (59.2)	32 (49.5)
refractory	20 (13.2)	6 (9.2)
Daratumumab		
naïve	131 (86.2)	63 (97)
exposed	13 (8.6)	1 (1.5)
refractory	8 (5.3)	1 (1.5)
Pomalidomide		
naïve	140 (92.1)	65 (100)
exposed	8 (5.3)	0 (0)
refractory	4 (2.6)	0 (0)
Prior ASCT		
yes	53 (34.9)	23 (35.4)
no	99 (65.1)	42 (64.6)
Reason for ixazomib initiation		
Clinical progression	58 (38.2)	26 (40)
Biochemical progression	81 (53.3)	31 (47.7)
Toxicity of previous therapy	13 (8.5)	8 (12.3)

## Data Availability

The original contributions presented in this study are included in the article. For further inquiries, please contact the corresponding author.

## Supplementary Materials

The following supporting information can be downloaded at: https://www.mdpi.com/article/10.3390/jcm15010286/s1, Figure S1: Eligibility and exclusion criteria; Figure S2: (A) Naive cross-study comparison of Progression Free Survival with ixazomib, lenalidomide, dexamethasone (IRd) and daratumumab, lenalidomide, dexamethasone (DRd) in Hungarian real-world patients; (B) Overall Survival with ixazomib, lenalidomide, dexamethasone (IRd) and daratumumab, lenalidomide, dexamethasone (DRd).

## Abbreviations

The following abbreviations are used in this manuscript:

AEs	adverse events
ASCT	autologous stem cell transplantation
DRd	daratumumab, lenalidomide, and dexamethasone
FISH	fluorescent in situ hybridization
FLC	free light chain
IMWG	international myeloma working group
IRd	ixazomib, lenalidomide, and dexamethasone
ISS	international staging system
KRd	carfilzomib, lenalidomide, dexamethasone
PFS	progression-free survival
RRMM	relapsed and/or refractory multiple myeloma
ORR	overall response rate
OS	overall survival
