# Supplementary material for: Ixazomib-Lenalidomide-Dexamethasone for the Treatment of Relapsed/Refractory Multiple Myeloma: A Hungarian Real-World Analysis
Source: J Clin Med. 2025 Dec 30;15(1):286. doi: 10.3390/jcm15010286 (PMC12786464; doi:10.3390/jcm15010286)
Supplement: Supplementary file 1 [file jcm-15-00286-s001.zip › jcm-4049127-supplementary.pdf]

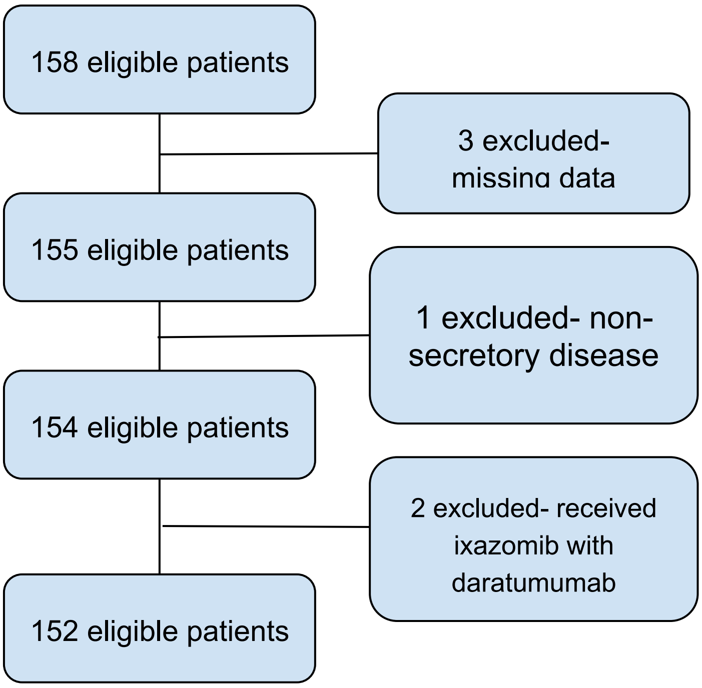

Supplementary Figure S1. Eligibility and exclusion criteria.

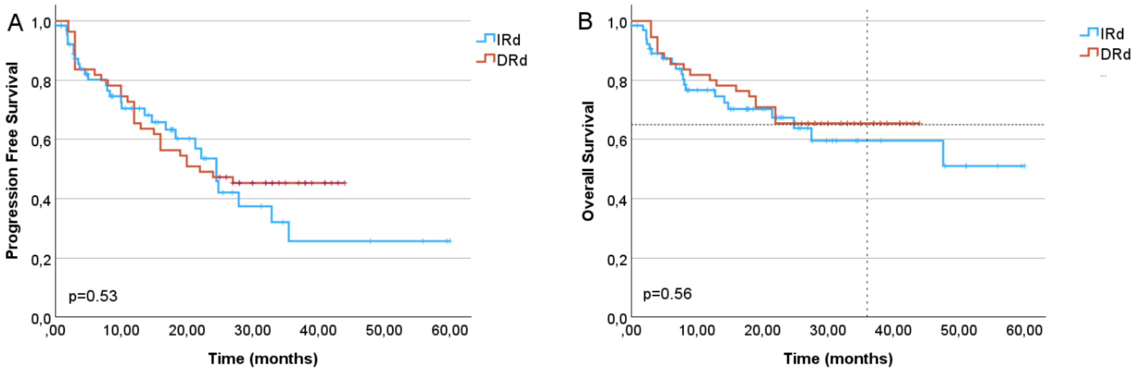

Figure S2. (**A**) Naive cross-study comparison of Progression Free Survival with ixazomib, lenalidomide, dexamethasone (IRd) and daratumumab, lenalidomide, dexamethasone (DRd) in Hungarian real-world patients; (**B**) Overall Survival with ixazomib, lenalidomide, dexamethasone (IRd) and daratumumab, lenalidomide, dexamethasone (DRd).
